# Epinephrine modulates Na^+^/K^+^ ATPase activity in Caco-2 cells via Src, p38MAPK, ERK and PGE2

**DOI:** 10.1371/journal.pone.0193139

**Published:** 2018-02-21

**Authors:** Layla El Moussawi, Mohamed Chakkour, Sawsan I. Kreydiyyeh

**Affiliations:** Department of Biology, Faculty of Arts & Sciences, American University of Beirut, Beirut, Lebanon; Universidade Federal do Rio de Janeiro, BRAZIL

## Abstract

Epinephrine, a key stress hormone, is known to affect ion transport in the colon. Stress has been associated with alterations in colonic functions leading to changes in water movements manifested as diarrhea or constipation. Colonic water movement is driven by the Na^+^-gradient created by the Na^+^/K^+^-ATPase. Whether epinephrine acts via an effect on the Na^+^/K^+^-ATPase hasn’t been studied before. The aim of this work was to investigate the effect of epinephrine on the Na^+^/K^+^-ATPase and to elucidate the signaling pathway involved using CaCo-2 cells as a model. The activity of the Na^+^/K^+^-ATPase was assayed by measuring the amount of inorganic phosphate released in presence and absence of ouabain, a specific inhibitor of the enzyme. Epinephrine, added for 20 minutes, decreased the activity of the Na^+^/K^+^-ATPase by around 50%. This effect was found to be mediated by α2 adrenergic receptors as it was fully abolished in the presence of yohimbine an α2-blocker, but persisted in presence of other adrenergic antagonists. Furthermore, treatment with Rp-cAMP, a PKA inhibitor, mimicked epinephrine’s negative effect and didn’t result in any additional inhibition when both were added simultaneously. Treatment with indomethacin, PP2, SB202190, and PD98059, respective inhibitors of COX enzymes, Src, p38MAPK, and ERK completely abrogated the effect of epinephrine. The effect of epinephrine did not appear also in presence of inhibitors of all four different types of PGE2 receptors. Western blot analysis revealed an epinephrine-induced increase in the phosphorylation of p38 MAPK and ERK that disappeared in presence of respectively PP2 and SB2020190. In addition, an inhibitory effect, similar to that of epinephrine’s, was observed upon incubation with PGE2. It was concluded that epinephrine inhibits the Na^+^/K^+^-ATPase by the sequential activation of α2 adrenergic receptors, Src, p38MAPK, and ERK leading to PGE2 release.

## Introduction

Stress, whether physical or mental, is a ubiquitous condition that is part of our everyday life. When confronted with potential stressors, the brain triggers a cascade of physiological reactions, known as the ‘‘fight or flight response”, to ensure the individual’s survival and adaptation to the threatening events [1]. Neural inputs from the brain stimulate the hypothalamus to release CRH (corticotrophin releasing hormone) which, in turn, activates both the sympathetic-adrenal medulla and pituitary-adrenal cortex axes, resulting in the respective release of the primary stress hormones: epinephrine and cortisol into the blood stream[2]. Together these hormones trigger the physiological deviations from homeostasis observed in the different systems of the body (cardiovascular, immune, endocrine, reproductive, respiratory, etc.…) during the acute stress response [1]. A key target of the stress reaction appears to be the gastrointestinal tract (GI) whereby the prevalence and the severity of several GI disorders were found to correlate with anxiety, depression, and neuroticism [3]. Among the various GI diseases, the role of stress in the pathophysiology of irritable bowel syndrome (IBS) has been extensively studied [4]. IBS is considered one of the most prominent chronic gastrointestinal disorders, and is mainly characterized by abdominal pain and discomfort due to either frequent diarrhea or constipation [5]. Epinephrine, a key stress hormone, was reported to affect water movement across the epithelium of certain tissues such as the human eye [6,7], lungs [8], and kidneys [9]. Nonetheless, a potential role of epinephrine, in the alteration of colonic water movement and the development of IBS symptoms, or even their exacerbation, has not been investigated before.

Water movement across epithelial layers of the colon is governed by the Na^+^ gradient created by the Na^+^/K^+^-ATPase. By pumping 3Na+ions to the outside of the cell in exchange for 2K ^+^ ions to the inside, the Na^+^/K^+^-ATPase establishes and maintains a low intracellular Na^+^ concentration which drives Na^+^ ions to flow down their electrochemical gradient from the lumen into the cytosol. This Na^+^ diffusion generates osmotic forces that cause water molecules to follow across the plasma membrane. Consequently, an alteration in the activity of the Na^+^/K^+^-ATPase was found to modify the direction and rate of net water transport, as detected in the intestines of deoxycorticosterone acetate- injected mice [10], in the ileum of methylprednisolone -pretreated rats [11], in rat proximal tubular cells following high Na^+^-diet [12], and in rat brain during acute cerebral ischemia [13].

In an attempt to understand the relation between the stress reaction and colonic water movement, we aimed to study the effect of epinephrine on the activity of the Na^+^/K^+^-ATPase in colon adenocarcinoma cells (CaCo-2), and to elucidate its underlying mechanism of action. Identifying the different mediators involved in the effect of epinephrine on the ATPase would help in finding therapeutic strategies that target them and relieve the undesirable effects of stress on colonic functions.

## Materials and methods

### Materials

Dulbecco’s Minimal Essential Medium (DMEM) with 4500mg glucose/L and pyridoxine HCl, Fetal Bovine Serum(FBS), Trypsin-EDTA, Penicillin/Streptomycin(PS), 10x Phosphate Buffered Saline (PBS) without calcium and magnesium, (-)-Epinephrine, L-Ascorbic Acid, N6,2′-O-Dibutyryladenosine 3′,5′-cyclic monophosphate sodium salt (dbcAMP), Adenosine 5′-triphosphate disodium salt(ATP), ouabain, prostaglandin E2 (PGE2), SC 19220, indomethacin, DL-propranolol, yohimbine, prazosin, and PF-04418948 were purchased from Sigma,Chemical Co,St. Louis Missouri, USA. PP2, PD98059 and SB202190, respective inhibitors of Src, MEK/ERK and p38MAPK, were obtained from Merck Millipore, MA, USA.

The human colon carcinoma cell line (CaCo-2) was purchased from American Type Culture Collection (ATCC), VA, USA.

Rabbit anti-ERK1/2 polyclonal antibody and rabbit anti-p-p44/42 MAPK (ERK1/2) monoclonal antibody were purchased respectively from Promega, WI, USA, and Cell Signaling, MA, USA. Rabbit anti-p38α, anti-p-p38α polyclonal antibodies, anti-rabbit IgG horse raddish peroxidase (HRP) conjugated, L-798106 and GW 627368X were purchased from Santa Cruz, CA, USA. Rabbit anti-Src and anti-pSrc polyclonal antibodies as well as anti GAPDH monoclonal antibody were purchased from Cell signaling, MA, USA. Protease inhibitors cocktail tablets were purchased from Boehringer Mannheim, Germany. Clarity ECL Substrate, Nitrocellulose membranes and Bio-Rad protein assay reagent were purchased from Bio-Rad, California, USA.

All other chemicals were purchased from Sigma, Chemical Co, St. Louis Missouri.

### Methods

#### Cell culture of CaCo-2 cells

CaCo-2 cells were used at passages 25–32. They were grown, at a density of 1200,000/well, on 100mm culture dishes in DMEM containing 4500 mg L-1 Glucose, sodium pyruvate, 1% Penicillin (100 μg mL-1), streptomycin (100 μg mL-1), 10% FBS, in a humidified incubator (95% O2, 5% CO2) at 37°C. Cells were always treated at 80–90% confluence.

#### Effect of epinephrine on the activity of the Na^+^/K^+^-ATPase

Dose and time response studies were conducted. Caco-2 cells were treated with epinephrine for different time intervals (0; 10; 20; 45;75 min) and at different concentrations (0; 0.05; 0.2; 0.5; 0.8 mM). Epinephrine was dissolved in 0.5M ascorbic acid. The positive and negative control groups were incubated with and without ascorbic acid respectively.

#### Protein extraction and determination

At the end of the treatment period, cultured cells were washed twice with PBS, lysed with histidine lysis buffer containing protease inhibitors, scraped, and homogenized at 4°C in a polytron at 22,000rpm. Proteins were quantified colorimetrically at a wavelength of 595nm using the Bradford Biorad assay.

#### Na^+^/K^+^-ATPase activity assay

The activity of the Na^+^/K^+^ ATPase was assayed as described by Esmann [14]. Protein concentration of the homogenate was adjusted to 0.5μg/μl using histidine buffer (150mM, pH 7.4). Samples were incubated with 1% saponin, added at a ratio of 1:4(v/v,) and phosphatase inhibitor cocktail for 30 min at room temperature. The final concentration of the cocktail components was: 10.7 mM glycerophosphate, 10.7mM pyrophosphate. Aliquots were then drawn and incubated at 37°C for an additional 30 min in histidine buffer containing NaCl (121.5mM), KCl (19.6 mM,), MgCl2 (3.92 mM), adenosine tri-phosphate (2.94 mM), in presence or absence of ouabain (1.47 mM), a specific inhibitor of the ATPase. At the end of the incubation period, the reaction was stopped by addition of 50% trichloroacetic acid at a ratio of 1:10 (v)/v and the samples were spun at 3000g for 5 min. The amount of inorganic phosphate liberated in the supernatant was measured colorimetrically at 750 nM according to the method of Taussky and Shorr [15].

#### Determination of the type of adrenergic receptors involved

The type of adrenergic receptors mediating the effect of epinephrine on the pump was determined by pre-treating the cells, 20 minutes prior to the addition of epinephrine, with the following antagonists: 0.1 mM yohimbine (α2 adrenergic antagonist), 50 μM prazosin (α1 adrenergic antagonist), or 0.03mM propranolol (non-selective β-adrenergic blocker).

Since β adrenergic receptors are coupled to Gs and the α2 to Gi, the effect of dbcAMP (10μM, 20min), a permeable cAMP analogue and RpcAMP (30μM), a PKA inhibitor, was studied. The vehicle was always added to the control in the same amount and for the same time.

#### Involvement of PGE2

Previous studies implicated PGE2 in the epinephrine effect on colonic ion transport. The involvement of PGE2 was investigated by adding indomethacin (100μM), a COX-inhibitor to the cells, 20 min prior to the addition of epinephrine, and by treating the cells with exogenous PGE2 (1nM, 20min). The effect of epinephrine was also investigated in presence of inhibitors of all four different PGE2 receptors, namely: PF-0441848 (1μM, EP2 antagonist), GW 627368X (10 μM, EP4 antagonist), SC 19220 (100 μM, EP1 antagonist), and L-798106 (1μM, EP3 antagonist). The inhibitors were added 20 min before epinephrine.

#### Involvement of Src

Adrenergic receptors were shown previously to activate the Src family of tyrosine kinases [16, 17]; hence the involvement of Src in the effect of epinephrine on the ATPase was investigated. The activity of the Na^+^/K^+^ ATPase was assayed in Caco-2 cells pre-incubated, 15 min before epinephrine, with PP2 (20μM), a Src inhibitor. Changes in the expression of phosphorylated Src at Tyr 416 were also studied by western blot analysis.

#### Involvement of p38MAPK and ERK

G protein coupled receptors have been reported to activate mitogen activated protein kinases [18]. To investigate if p38MAPK and ERK have any role to play in the effect of epinephrine on the pump, Caco-2 cells were treated, 15 min before epinephrine with SB 202190 (50 μM), a specific inhibitor of p38MAPK, or with PD98059 (50 μM) a MEK/ERK specific inhibitor.

Changes in the level of phosphorylated ERK and p38MAPK, the active forms of the kinases, were also examined by western blot analysis.

#### Locating the different intermediates with respect to PGE2 and with respect to each other

To determine if each of Src, p38MAPK and ERK is upstream or downstream of PGE2, the cells were treated with epinephrine or PGE2 when each of these kinases was inhibited with respectively PP2, SB 20190 or PD98059.

The effect of epinephrine and PGE2 on the phosphorylation of each of cSrc (Tyr 416), p38MAPK and ERK was also investigated by western blotting.

#### Western blot analysis

Treated cells were lysed and homogenized in a polytron (20,000–22,000 rpm) at 4°C after addition of a cocktail of phosphatase inhibitors. Proteins were quantified using the Bradford method. Equal amounts of proteins (40 μg) were loaded and resolved on 10% SDS polyacrylamide gel and transferred to a nitrocellulose membrane which was then blocked and incubated with a primary ERK1/2, p- ERK1/2, p38α, p -p38α, Src or p-Src antibody, followed by an incubation with a goat anti-rabbit secondary horse raddish peroxidase (HRP) conjugated IgG. The signal was detected by chemiluminescence using Clarity ECL Substrate. The intensity of the signal was detected using a ChemiDoc imager. After probing with an antibody for a phosphorylated kinase, the membrane was sequentially stripped and reprobed with an antibody for the unphosphorylated form of the kinase and then with an antibody for GAPDH. The bands for the phosphorylated kinases were normalized to their total unphosphorylated kinases using Image lab software.

#### Statistical analysis

Results are reported as means ± SEM and are tested for statistical significance by a one-way Analysis of Variance (ANOVA) followed by Tukey-Kramer multiple comparisons test using Instat and Excel Softwares.

## Results

### Dose and time response study on the effect of epinephrine on the Na^+^/K^+^-ATPase

**Fig 1:**
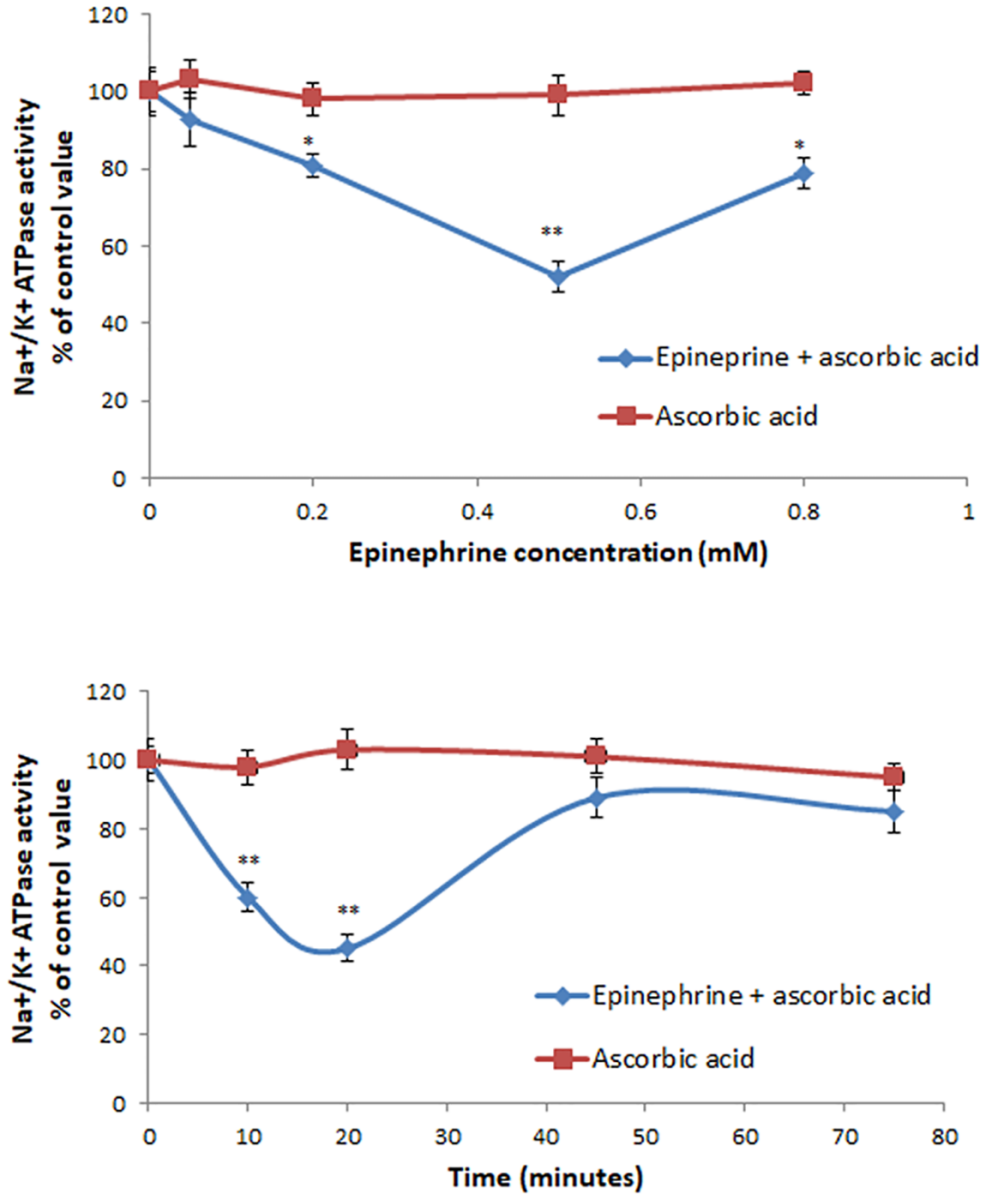
Dose (A) and time (A) response study on the effect of epinephrine on the Na^+^/K^+^ ATPase activity. Values are means ±SEM of a minimum of 3 observations. * Significantly different from the control at P<0.05. ** Significantly from the control at P<0.01.

Epinephrine (dissolved in ascorbic acid 0.5M) reduced in a dose and time-dependent manner the activity of the Na^+^/K^+^ ATPase in Caco-2 cells. The highest inhibitory effect was observed at 20 min and at a dose of 0.5 mM. Accordingly in all other experiments cell were treated with epinephrine for 20min and at a concentration of 0.5mM. Ascorbic acid alone exerted no significant effect on the activity of the pump (Fig 1).

### Epinephrine acts via alpha-2 adrenergic receptors

**Fig 2:**
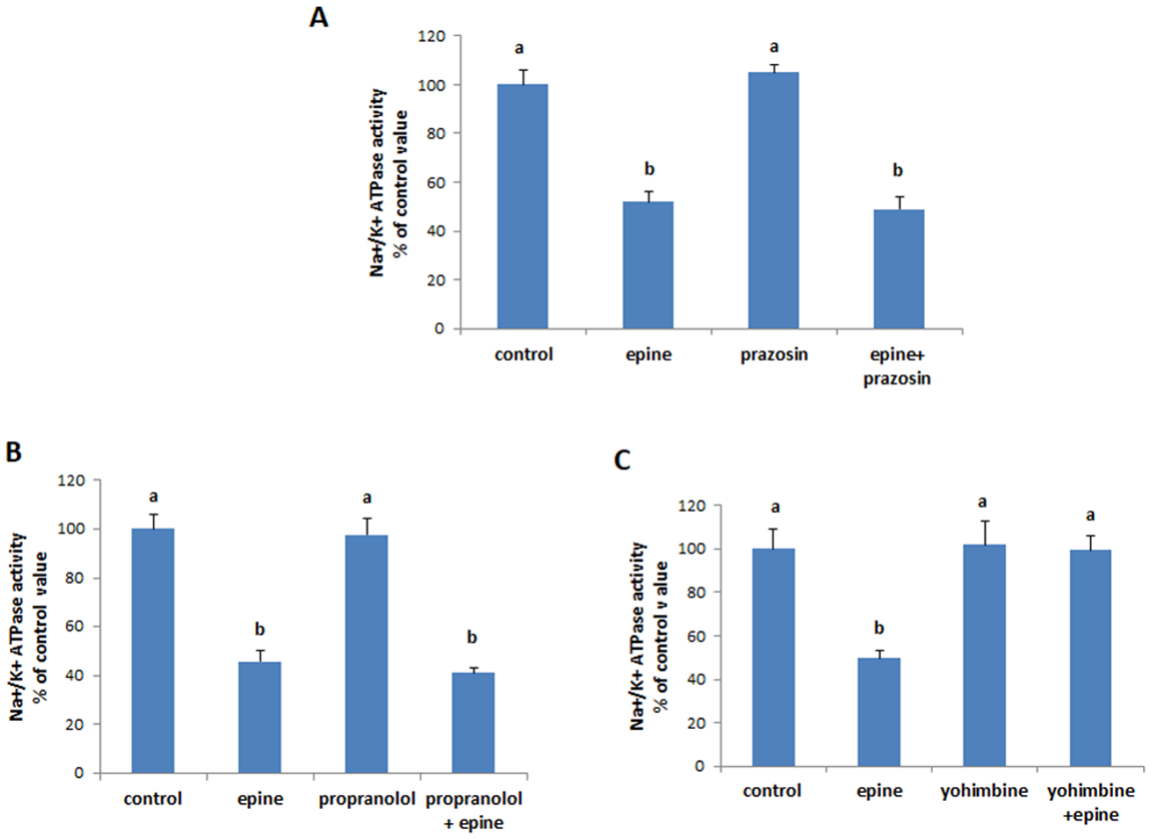
Epinephrine inhibits the Na^+^/K^+^ ATPase by activating α2 adrenergic receptors. Effect of epinephrine (0.5 mM, 20min) on the Na^+^/K^+^ ATPase activity in presence of (A) prazosin (50 μM), an α1 blocker (B) propranolol (0.03M), a β adrenergic blocker, and (C) yohimbine (0.1mM), an α2 blocker. Blockers were added 20 min prior to the addition of epinephrine. Values are means ±SEM of a minimum of 3 observations. Bars not sharing a common letter are significantly different from each other at P<0.01.

The inhibitory effect of epinephrine on the Na^+^/K^+^-ATPase persisted when the cells were pre- incubated with of 0.03 mM propranolol (non selective β-adrenergic blocker) or 50 μM prazosin (selective α1 antagonist), but was no longer apparent in the presence of 0.1 mM yohimbine (Fig 2), a selective α2- adrenergic antagonist, suggesting that epinephrine exerts its effect by exclusively binding to its α2-adrenergic receptors.

### Epinephrine inhibits PKA

**Fig 3:**
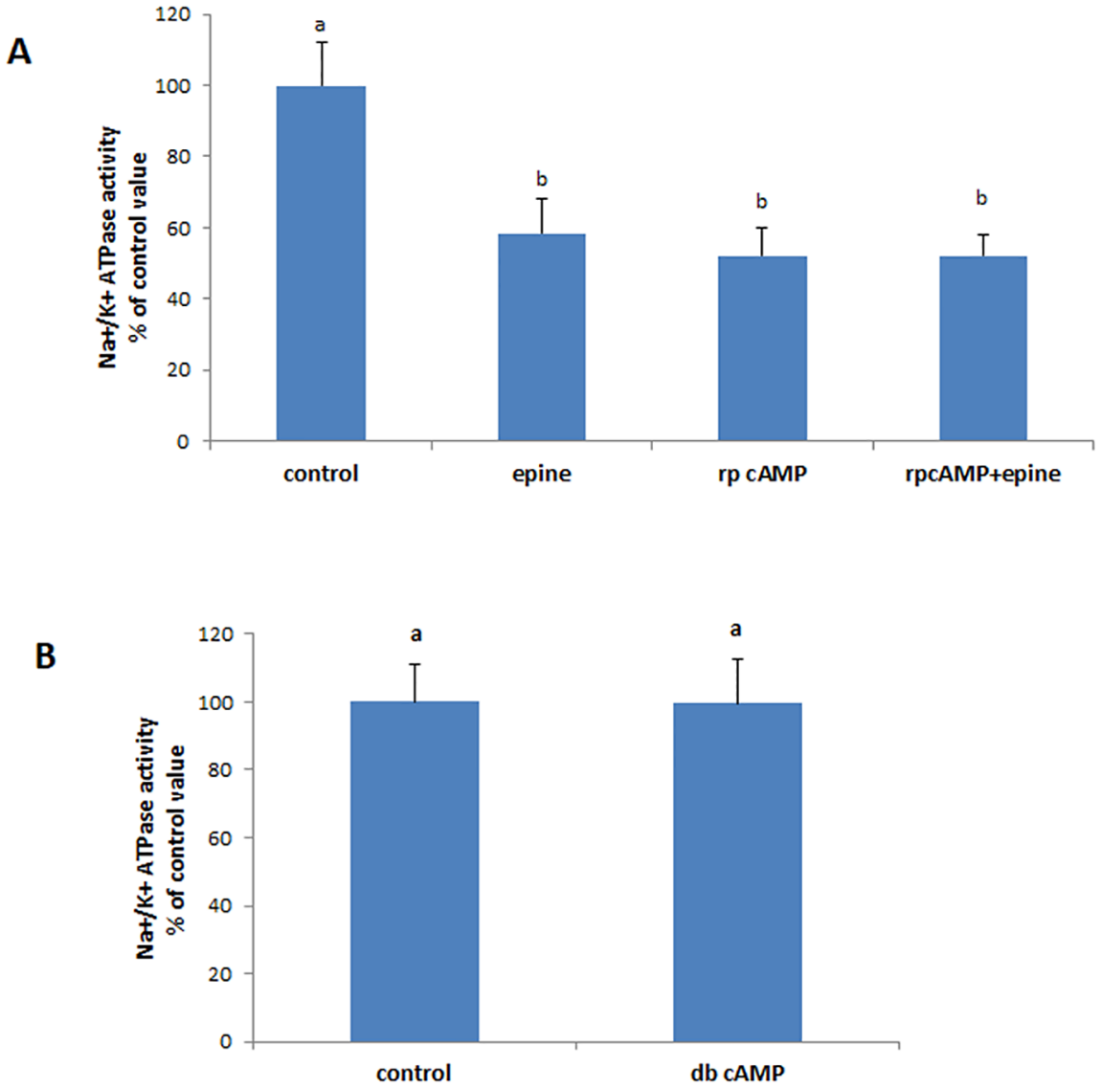
(A)RpcAMP (30 μM, 20min), an inhibitor of PKA, reduced the Na^+^/K^+^ ATPase activity while (B) dbcAMP (10μM, 20min), had no effect. Values are means ±SEM of 3 observations. Bars not sharing a common letter are significantly different from each other at P<0.01.

It is well known that α2-adrenergic receptors are coupled to Gi which acts to down-regulate cAMP and inhibit PKA. Treating the cells with RpcAMP (30μM), a cell permeable PKA inhibitor alone, mimicked the inhibitory effect of epinephrine on the Na^+^/K^+^-ATPase, and didn’t result in any additive inhibition when added simultaneously with epinephrine (Fig 3A). The cell permeable cAMP analogue dbcAMP, did not have however, any effect on the ATPase (Fig 3B).

### Determination of the mediators involved

#### PGE2

**Fig 4:**
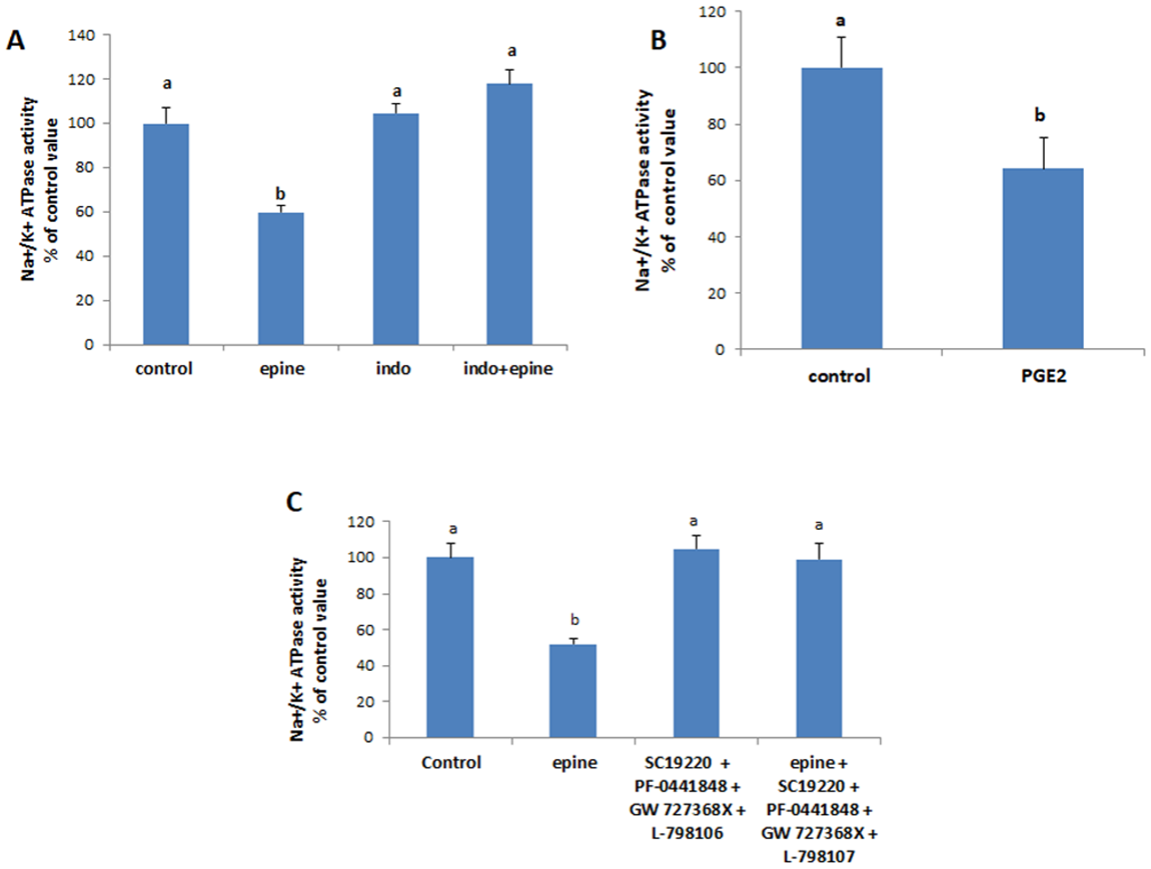
Involvement of PGE2 in epinephrine signaling. (A) Effect of epinephrine (0.5 mM, 20min) on the activity of the Na^+^/K^+^ ATPase in presence of indomethacin (100μM, a COX inhibitor added 20 min before epinephrine; (B) Effect of exogenous PGE2 (1nM,20min) on pump’s activity. (C) Effect of epinephrine in presence of inhibitors of all PGE2 receptors. Values are means ± SEM of 4 observations. Bars not sharing the same letter are considered significantly different from each other at p<0.01.

To test the possibility that epinephrine might be signaling through PGE2, CaCo-2 cells were treated with epinephrine in presence of indomethacin, an inhibitor of COX enzymes. The inhibitor abolished the effect of epinephrine and restored the activity of the Na^+^/K^+^-ATPase back to control levels (Fig 4A). The effect of epinephrine was not manifested when all four PGE2 receptors were blocked with selective antagonists (Fig 4C). Indomethacin alone and the antagonists alone didn’t cause any significant change in the activity of the Na^+^/K^+^-ATPase.

To confirm the involvement of PGE2 in the effect of epinephrine, Caco-2 cells were treated with exogenous PGE2. The prostaglandin exerted a similar inhibitory effect to that observed with epinephrine (Fig 4B).

#### C-Src

**Fig 5:**
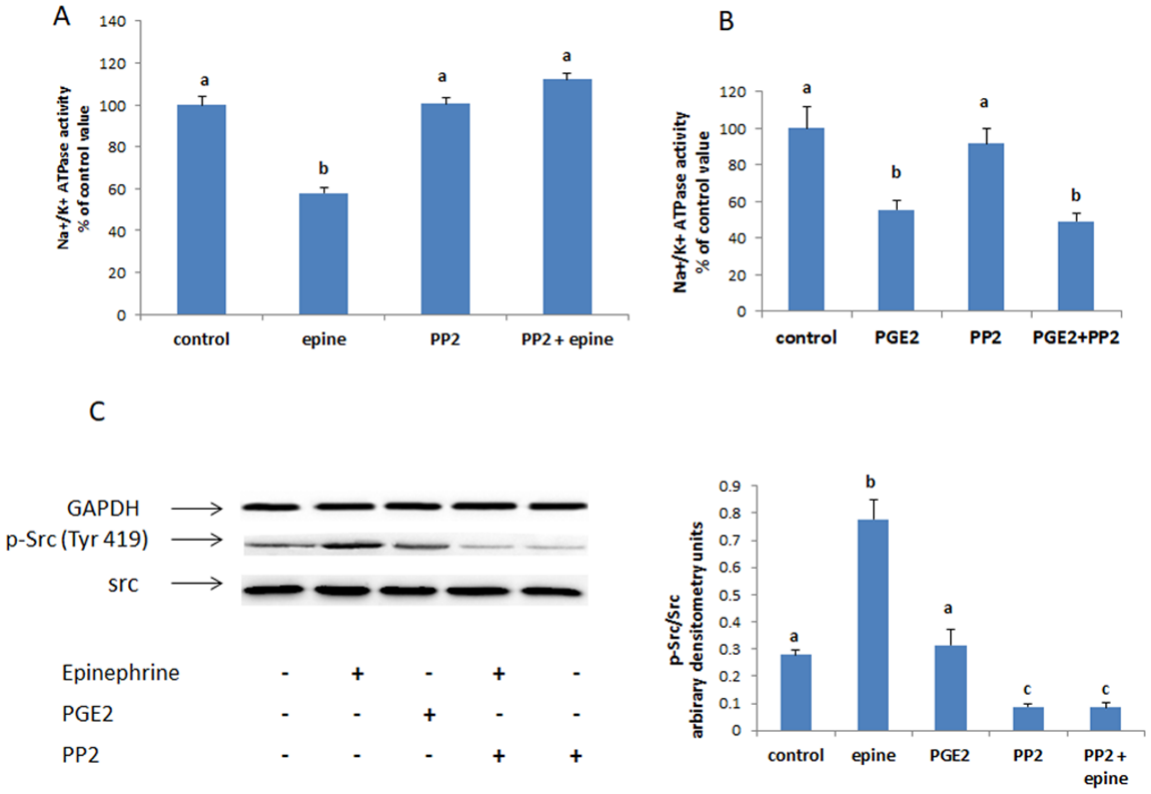
(A) Effect of epinephrine (0.5 mM, 20min) and (B) PGE2 (1nM,20min) on the activity of the Na^+^/K^+^ ATPase in presence of the Src inhibitor PP2 (20μM, added 15 min before epinephrine or PGE2). Values are means ± SEM of 3 observations. Bars not sharing the same letter are considered significantly different from each other at p<0.01. (C) Epinephrine but not PGE2 increased Src phosphorylation. Values of the normalized data (p-Src/Src) are means ± SEM of 3 observations. Densitometry bars that do not share the same letter are considered significantly different from each other at p<0.01. The blots are representative of an experiment repeated 3 times.

In presence of the specific inhibitor of c-Src, PP2, the effect of epinephrine disappeared (Fig 5A) but that of PGE2 was maintained (Fig 5B). Similarly, western blot analysis showed a significant increase in the phosphorylation of c-Src(Tyr 416) by epinephrine but not by PGE2(Fig 5C). The expression of un-phosphorylated Src was not affected.

#### P38MAPK

**Fig 6:**
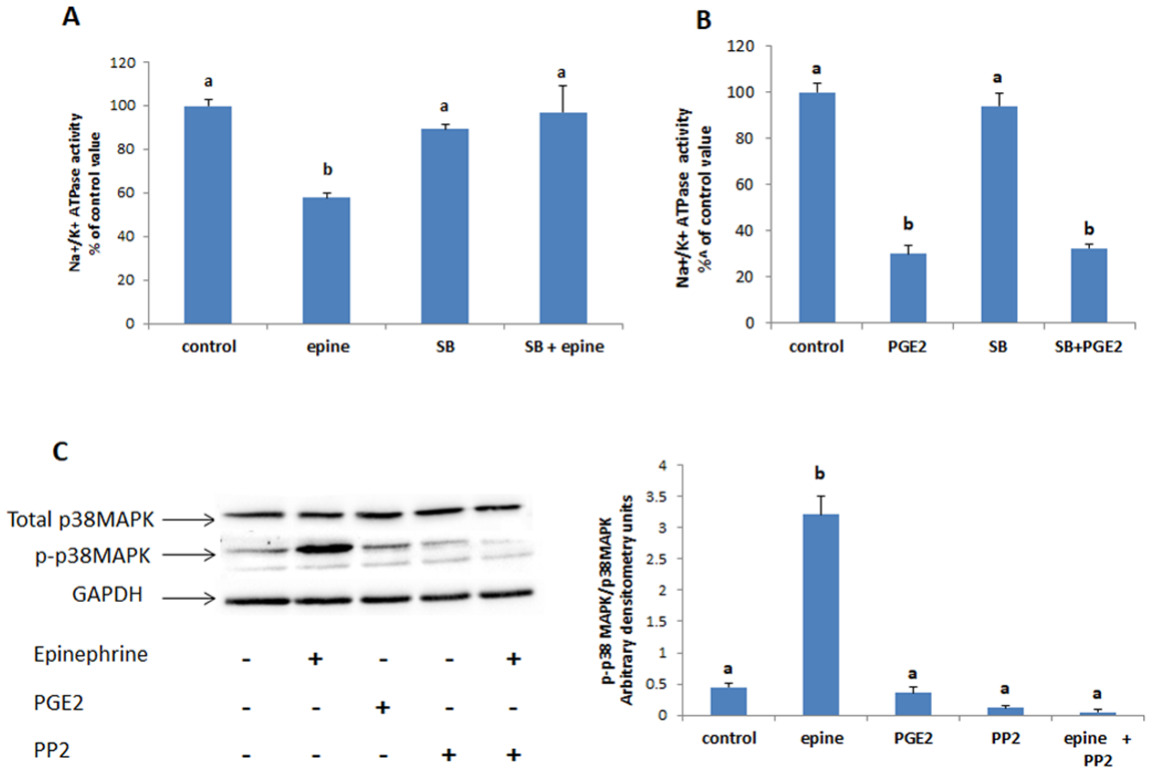
(A) Effect of epinephrine (0.5 mM, 20min) and (B) PGE2 (1nM,20min) on the activity of the Na^+^/K^+^ ATPase when p38MAPK was inhibited with SB202190 (50μM, added 15 min before epinephrine or PGE2). Values are means ± SEM of 3 observations. Bars not sharing the same letter are considered significantly different from each other at p<0.01. (C)Epinephrine increased significantly the phosphorylation of p38MAPK but PGE2 exerted no effect. Values of the normalized data (p38MAPK / p38MAPK) are means ± SEM of 3 observations. Densitometry bars that do not share the same letter are considered significantly different from each other at p<0.01. The blots are representative of an experiment repeated 4 times.

Similarly, inhibition of p38MAPK with SB 202190 obliterated the effect of epinephrine on the ATPase (Fig 6A), but not that of PGE2 (Fig 5B). Western blot analysis showed also a very significant increase in the phosphorylation of p38MAPK that disappeared in presence of PP2 (Fig 5C).

#### ERK

**Fig 7:**
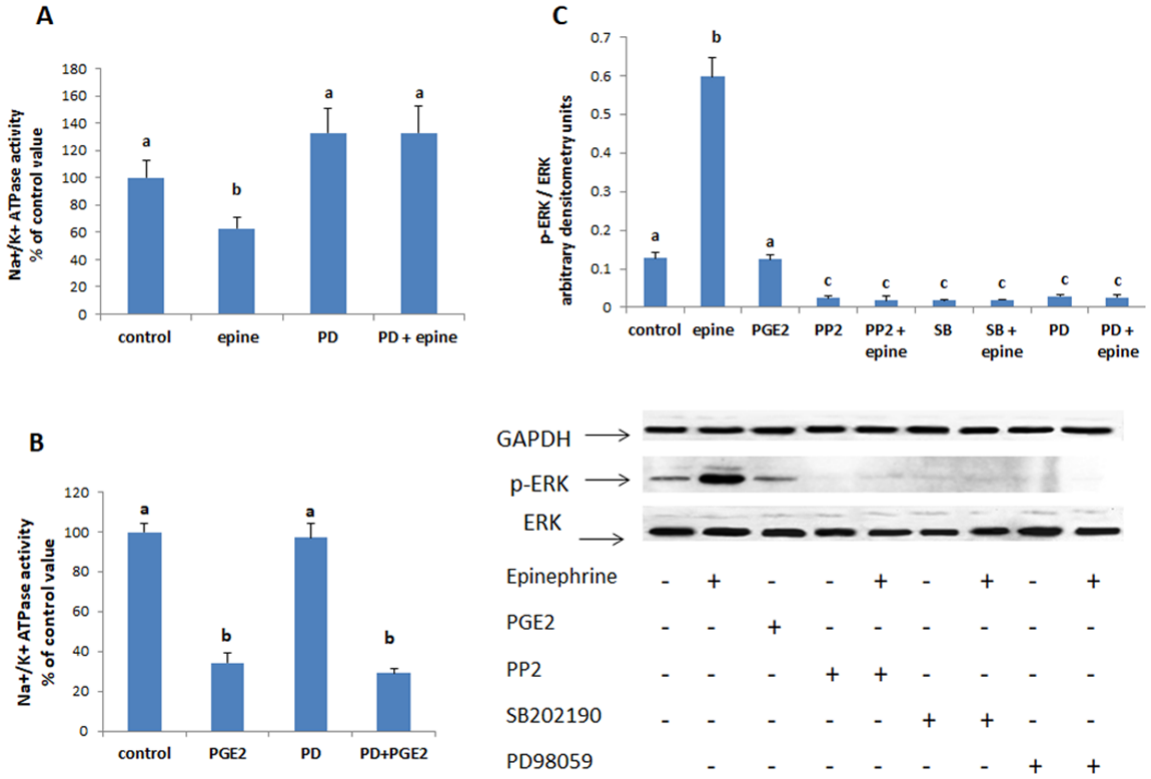
(A) Effect of epinephrine (0.5 mM, 20min) and (B) PGE2 (1nM, 20min) on the activity of the Na^+^/K^+^ ATPase when ERK was inhibited with PD98059 (50μM, added 15 min before epinephrine or PGE2). Values are means ± SEM of 3 observations. Bars not sharing the same letter are considered significantly different from each other at p<0.01. (C) Epinephrine but not PGE2 increased significantly the phosphorylation of ERK. Values of the normalized data (p-ERK/ERK) are means ± SEM of 3 observations. Densitometry bars that do not share the same letter are considered significantly different from each other at p<0.01. The blots are representative of an experiment repeated 3 times.

The specific inhibitor of ERK, PD98059, suppressed the effect of epinephrine (Fig 7A) but maintained that of PGE2 (Fig 7B). There was also an increase in the phosphorylation of ERK by epinephrine but not by PGE2 (Fig 7C). This increase disappeared in presence of PP2 or SB202190. Epinephrine did not affect the protein expression of unphosphorylated ERK.

## Discussion

While norepinephrine has been established as a regulator of the Na^+^/K^+^-ATPase in different cells, like brain, kidney and arterial cells [19–21], very few studies investigated the role of its derivative epinephrine on intestinal cells. The scarce literature reports a stimulatory effect of epinephrine on the Na^+^/K^+^-pump in rat jejunal crypt cells [22] and skeletal muscles [23]. Its mechanism of action remains however till now ill-defined. This work studies the effect of epinephrine on the colonic ATPase using Caco-2 cells as a model and tries to elucidate the signaling pathway involved.

Treatment of CaCo-2 cells with epinephrine resulted in a decrease in the activity of the Na^+^/K^+^-ATPase that was dose and time dependent with a maximal inhibitory effect at a dose of 0.5mM and an incubation period of 20min. This dose and time dependency may be ascribed to differences in the affinity of the adrenergic receptors, to their desensitization with time, or to the production of signaling intermediates that exert themselves a time dependent effect. The inhibitory effect of epinephrine appears to be mediated via α2-adrenergic receptors exclusively, since it disappeared only in presence of yohimbine, the specific α2 adrenergic blocker, but persisted when epinephrine was simultaneously added with other adrenergic antagonists. Had other receptors been involved, then a partial inhibitory effect would still be observed in presence of yohimbine. Epinephrine’s preferential binding to α2-adrenergic receptors can be attributed to the α2 –receptors’ higher cell surface density, higher affinity to epinephrine, or both, when compared to other types of adrenergic receptors. The differential characteristics of adrenergic receptors in CaCo-2 cells haven’t been addressed before; nonetheless, α2- receptors, individually, were shown to be highly expressed in a similar adenocarcinoma cell line, HT-29, and to have the highest affinity to epinephrine among other adrenergic agonists [24].

In accordance with the widely accepted notion that α2-adrenergic receptors are coupled to inhibitory G-proteins (Gi) and act to down-regulate the production of cAMP and consequently PKA activity [25], cells treated with RpcAMP, a PKA inhibitor exhibited a similar decrease in Na^+^/K^+^-ATPase activity as those treated with epinephrine, and the simultaneous addition of epinephrine and RpcAMP didn’t result in any additive effect. The data support thus an involvement of Gi in the response to epinephrine. Stimulating PKA with dbcAMP was without any effect on the ATPase.

PKA is known to modulate the Na^+^/K^+^ ATPase activity by direct phosphorylation or indirectly through activation of other mediators. PKA phosphorylation at Ser 943 was first identified in the C-terminal of the rat renal Na^+^/K^+^-ATPase α1 subunit [26]. The effects of this phosphorylation in various cell types varies between activation [27], inhibition [28], or no change at all [26]. PKA in this work seems to have a stimulatory effect and is needed for the basal activity of the ATPase since its inhibition with RpcAMP resulted in a lower ATPase activity.

Alpha-2 adrenergic receptors were reported to signal via PGE2 in various tissues.

The prostaglandin was found to be responsible for the α2 adrenoceptor-dependent urea transport and hyperthermic response in rat IMCD [29] and guinea pigs [30] respectively. Activation of α2 adrenergic receptors in cutaneous cells induced also PGE2 release [31].

On the other hand, PGE2 is a recognized regulator of the Na^+^/K^+^-ATPase activity. Its inhibitory effect was observed in several organs including heart [32], liver [33, 34], and kidneys [35]. This work revealed also an inhibitory effect of the prostaglandin in colonic cells, which was behind the epinephrine-induced decrease in the Na^+^/K^+^ ATPase activity, since the effect of the hormone on the ATPase was suppressed when the synthesis of PGE2 was blocked with indomethacin.

The inhibitory effect of epinephrine on the Na^+^/K^+^ ATPase was also abrogated in presence of PP2, an inhibitor of the cytoplasmic tyrosine kinase Src, but that of PGE2 was maintained, implying that Src is involved in epinephrine’s signaling and is upstream of PGE2. Src activity is regulated by phosphorylation. While phosphorylation at tyrosine Y527 inhibits the kinase by stabilizing a confirmation that prevents interaction with the substrate, phosphorylation at tyrosine Y416 increases the activity of Src by stabilizing the activation loop [36]. Western blot analysis showed a greater epinephrine but not PGE2-induced phosphorylation of Src (Tyr 416), confirming the involvement and position of Src in the signaling pathway. Such an involvement came as no surprise since G protein coupled receptors in general [37] and alpha-2 adrenergic receptors in particular, are known to increase the activity of Src kinase in a pertussis toxin sensitive manner, inferring that this increase is mediated via a Gi protein [38]. Src is usually maintained in an inactive state by phosphorylation of the C-terminal regulatory tyrosine by C-terminal Src kinase (Csk). Abrahamsen et al. [39] demonstrated that PKA increases the activity of Csk and consequently represses Src. The stimulation of Src observed in this work may be due the Gi- induced inhibition of PKA which in turn decreases the activity of Csk and relieves Src of its inhibitory effect.

We identified in addition, p38MAPK as another mediator of epinephrine signaling. In presence of SB202190, a specific inhibitor of the kinase, the effect of epinephrine on the Na^+^/K^+^ pump did not appear, while that of PGE2 was still observed, suggesting that p38MAPK is upstream of PGE2. This conclusion was supported by the western blots which showed a very significant increase in the phosphorylation of p38MAP by epinephrine. This increase was not manifested when Src kinase was inhibited with PP2, indicating that Src is upstream of p38MAPK. The findings are in line with those of Thobe et al. [40] who demonstrated in mice Kupffer cells, an activation of p38MAPK in response to hypoxia that was induced by Src activation. Similarly TGFβ1 was shown by Pechkovsky et al. [41] to increase αvβ3 integrin expression in human lung fibroblasts through a c-Src dependent activation of p38MAPK. Activation of p38 by Src was also shown to be required for EGF-stimulated intestinal epithelial monolayer restitution [42].

Another MAPK found to be involved in the inhibitory effect of epinephrine is ERK: The activity of the ATPase was restored back to control values in cells treated with epinephrine in presence of PD98059, an inhibitor of MEK/ERK. The inhibitory effect of PGE2 was, however, not affected by ERK inhibition, inferring that ERK works upstream of PGE2. Western blot analysis showed also a significant increase in ERK phosphorylation upon treatment with epinephrine but not upon treatment with PGE2. The epinephrine—induced increase in p-ERK expression did not appear when Src and p38 MAPK were inhibited respectively with PP2 and SB202190, implying that ERK is downstream of p38MAPK which is downstream of Src. Whether p38MAPK activates ERK by cross-talking directly or indirectly with one or more of the components of the ERK module is not clear and needs further investigation. Up to our knowledge, no previous work reported a p38MAPK dependent activation of ERK. The interactions reported in the literature between the two kinases are of a negative nature [43] and mediated via the protein phosphatase 2A [44].

Sine ERK is upstream of PGE2, it may play a role in its production. PGE2 synthesis is catalyzed by the COX enzymes, COX-2 being the inducible isoform. The activation of COX-2 by ERK has been frequently reported. Chae et al. [45] showed that inhibition of ERK pathway blocked the TNF-α induced PGE2 release. ERK was shown also to mediate the increase in COX-2 expression induced by thrombin in human lung fibroblasts [46] as well as the PGE2 release responsible for neuronal death in TDP-43-depleted microglia [47] and the salicylate induced increase in COX-2 expression in osteoblasts [45].

PGE2 appears to act as a negative modulator of the pump in a variety of tissues. An increase in PGE2, associated with a decrease in cAMP, mediated angiotension II inhibitory effects on Na^+^/K^+^-ATPase and water absorption in rat jejunum [48]. PGE2 was also shown to reduce Na^+^/K^+^-ATPase protein expression in LLCPK1 [49], cardiomyocytes [32], and HepG2 cells [33]. Incubation of rat hippocampus, both *in vivo* and in vitro, with PGE2 for 30 min led to a dose dependent decrease in the Na^+^/K^+^-ATPase activity attributed to the PKA and PKC- dependent Ser943 phosphorylation of the α subunit [50].

It can be concluded that epinephrine inhibits the Na^+^/K^+^ ATPase in Caco-2 cells via α2 adrenergic receptors leading to the sequential activation of Src, p38MAP, ERK, COX-2 and eventually PGE2 release. The prostaglandin reduces the activity of the ATPase through another signaling pathway that needs to be explored.

**Fig 8:**
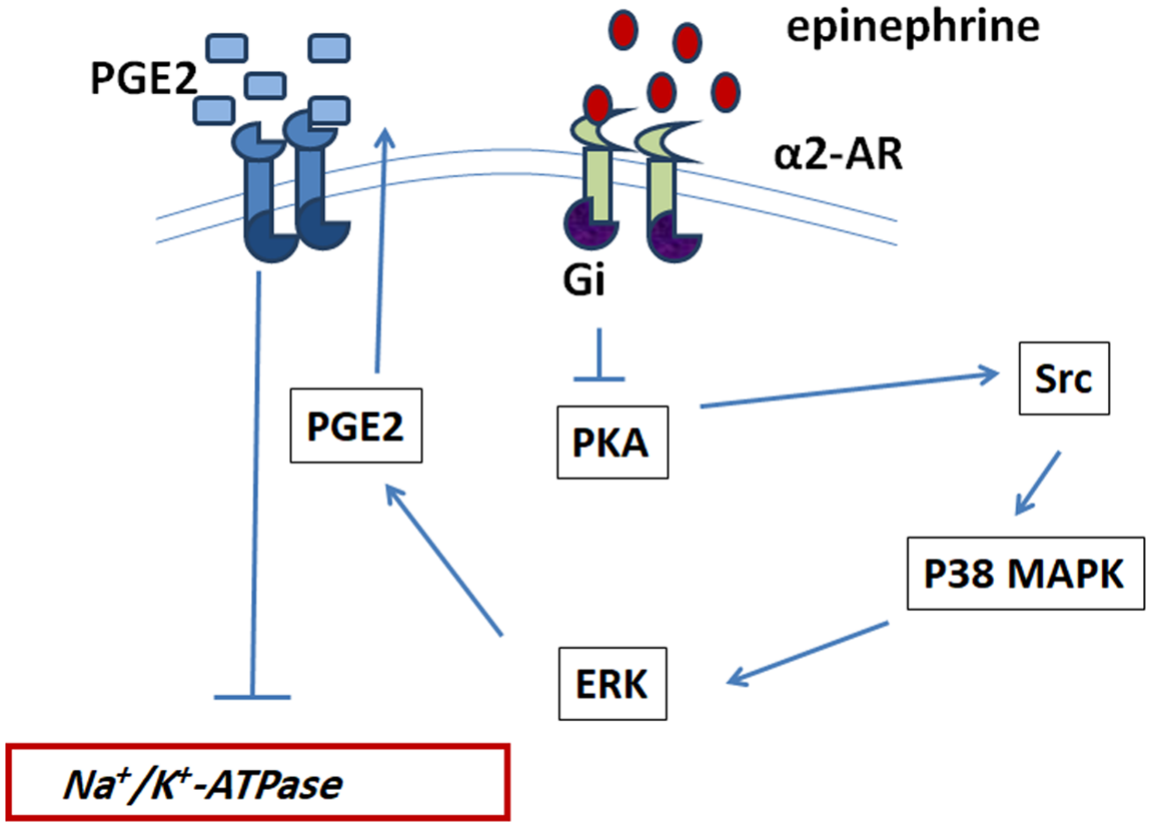
Proposed signaling pathway of epinephrine’s action on the Na+/K+ ATPase.

The pathway involved in the effect of epinephrine on the Na+/K+ ATPase is represented in Fig 8.

## References

[1] McEwen B. Central effects of stress hormones in health and disease: Understanding the protective and damaging effects of stress and stress mediators. European Journal of Pharmacology. 2008; 583(2–3):174–185. doi: 10.1016/j.ejphar.2007.11.071 18282566 10.1016/j.ejphar.2007.11.071 PMC2474765

[2] Kemeny M. The Psychobiology of Stress. Current directions in psychological science. 2003; 12 (4): 124–129.

[3] Bhatia V, Tandon R K (2005). Stress and the gastrointestinal tract. Journal of gastroenterology and hepatology. 2005; 20(3): 332–339. doi: 10.1111/j.1440-1746.2004.03508.x 15740474 10.1111/j.1440-1746.2004.03508.x

[4] Lee C, Doo E, Choi J, Jang S, Ryu H, Lee J et al The Increased Level of Depression and Anxiety in Irritable Bowel Syndrome Patients Compared with Healthy Controls: Systematic Review and Meta-analysis. Journal of Neurogastroenterology and Motility. 2017; 23(3):349–362. doi: 10.5056/jnm16220 28672433 10.5056/jnm16220 PMC5503284

[5] Everhart J, Renault P. Irritable bowel syndrome in office-based practice in the United States. Gastroenterology. 1991; 100(4):998–1005. 2001837 10.1016/0016-5085(91)90275-p

[6] Erickson-Lamy K, Nathanson J. Epinephrine increases facility of outflow and cyclic AMP content in the human eye in vitro. Invest Ophthalmol Vis Sci. 1992; 33: 2672–8. 1353486

[7] Nathanson J A, Scavone C, Scanlon C, McKee M. The cellular Na+ pump as a site of action for carbon monoxide and glutamate: A mechanism for long-term modulation of cellular activity. Neuron. 1995; 14(4): 781–794. 7718240 10.1016/0896-6273(95)90222-8

[8] Lane S, Maender K, Awender N, Maron M.Adrenal Epinephrine Increases Alveolar Liquid Clearance in a Canine Model of Neurogenic Pulmonary Edema. American Journal of Respiratory and Critical Care Medicine. 1998; 158(3):760–768. doi: 10.1164/ajrccm.158.3.9802031 9731002 10.1164/ajrccm.158.3.9802031

[9] Hawk C T, Kudo L H, Rouch A J, Schafer J A. Inhibition by epinephrine of AVP- and CAMP-stimulated Na+ and water transport in Dahl rat CCD. Am J Physiol. 1993; 265:F449–60. doi: 10.1152/ajprenal.1993.265.3.F449 8105698 10.1152/ajprenal.1993.265.3.F449

[10] Charney A, Kinsey M, Myers L, Gainnella R, Gots R. Na+-K+-activated adenosine triphosphatase and intestinal electrolyte transport. Effect of adrenal steroids. Journal of Clinical Investigation. 1975; 56(3):653–660. doi: 10.1172/JCI108135 125764 10.1172/JCI108135 PMC301913

[11] Charney A, Donowitz M. Prevention and reversal of cholera enterotoxin-induced intestinal secretion by methylprednisolone induction of Na+-K+-ATPase. Journal of Clinical Investigation. 1976; 57(6):1590–1599. doi: 10.1172/JCI108429 132458 10.1172/JCI108429 PMC436818

[12] Di Campo V, Henríquez LM, Proverbio T, Marín R, Proverbio F.Effect of a high Na+diet on cell volume and Na+-stimulated ATPase activities of rat kidney membranes. FEBS Letters. 1990; 274(1–2):96–98. 2174805 10.1016/0014-5793(90)81338-o

[13] Mintorovitch J, Yang G, Shimizu H, Kucharczyk J, Chan P, Weinstein P. Diffusion-Weighted Magnetic Resonance Imaging of Acute Focal Cerebral Ischemia: Comparison of Signal Intensity with Changes in Brain Water and Na+,K+ -ATPase Activity. Journal of Cerebral Blood Flow & Metabolism. 1994;14(2):332–336. 8113328 10.1038/jcbfm.1994.40

[14] Esman M. ATPase and phosphatase activity of Na+,K+-ATPase: molar and specific activity, protein determination. Methods in Enzymology.1988;156:105–109 2835596 10.1016/0076-6879(88)56013-5

[15] Taussky H, Shorr E. Microcolorimetric Method for Determination of Inorganic Phosphorous. J. Biol. Chem. 1953; 202: 675–685. 13061491

[16] Simonson MS, Herman WH. Protein kinase C and protein tyrosine kinase activity contribute to mitogenic signaling by endothelin-1. Cross-talk between G protein-coupled receptors and pp60c-src.J Biol Chem. 1993; 268(13):9347–57. 7683650

[17] Ishida M, Marrero M, Schieffer B, Ishida T, Bernstein K, Berk B. Angiotensin II Activates pp60c-src in Vascular Smooth Muscle Cells. Circulation Research. 1995; 77(6):1053–1059. 7586216 10.1161/01.res.77.6.1053

[18] Naor Z, Benard O, Seger R. Activation of MAPK Cascades by G-protein-coupled Receptors: The Case of Gonadotropin-releasing Hormone Receptor. Trends in Endocrinology & Metabolism. 2000; 11(3):91–99. 10707049 10.1016/s1043-2760(99)00232-5

[19] Ádám-Vizi V., Vizi E. S., & Horvath I. Stimulation by noradrenaline of Na+K+ ATPase in different fractions of rat brain cortex. Journal of Neural Transmission. 1979;46(1):59–69. 228003 10.1007/BF01243429

[20] Ohtomo Y, Meister B, Hökfelt T, Aperia A. Coexisting NPY and NE synergistically regulate renal tubular Na+, K+-ATPase activity. Kidney International. 1994; 45(6):1606–1613. 7523751 10.1038/ki.1994.211

[21] Pérez-Vizcaíno F, Cogolludo A, Tamargo J. Modulation of arterial Na+-K+-ATPase-induced [Ca2+] i reduction and relaxation by norepinephrine, ET-1, and PMA. American Journal of Physiology-Heart and Circulatory Physiology. 1999; 276(2): H651–H657. 10.1152/ajpheart.1999.276.2.H651 9950867

[22] Kreydiyyeh S. Epinephrine stimulates the Na+-K+ ATPase in isolated rat jejunal crypt cells. Life Sciences. 2000; 67(11):1275–1283. 10972196 10.1016/s0024-3205(00)00717-7

[23] James J H, Wagner K R, King J K, Leffler R E, Upputuri R K, Balasubramaniam A et al Stimulation of both aerobic glycolysis and Na+-K+-ATPase activity in skeletal muscle by epinephrine or amylin. American Journal of Physiology-Endocrinology And Metabolism. 1999; 277(1): E176–E186. 10.1152/ajpendo.1999.277.1.E176 10409142

[24] Bouscarel B, Cortinovis C, Carpene C, Murat J, Paris H. α2-Adrenoceptors in the HT 29 human colon adenocarcinoma cell line: Characterization with [3H] clonidine; Effects on cyclic AMP accumulation. European Journal of Pharmacology. 1985; 107(2):223–231. 2984004 10.1016/0014-2999(85)90062-7

[25] Taussig R, Iniguez-Lluhi J, Gilman A. Inhibition of adenylyl cyclase by Gi alpha. Science. 1993; 261(5118):218–221. 8327893 10.1126/science.8327893

[26] Feschenko M, Sweadner K. Structural Basis for Species-specific Differences in the Phosphorylation of Na,K-ATPase by Protein Kinase C. Journal of Biological Chemistry. 1995;270(23):14072–14077. 7775468 10.1074/jbc.270.23.14072

[27] Cornelius F, Logvinenko N. Functional regulation of reconstituted Na, K-ATPase by protein kinase A phosphorylation. FEBS Letters. 1996;380(3):277–280. 8601440 10.1016/0014-5793(96)00032-4

[28] Bertorello A, Aperia A, Walaas S, Nairn A, Greengard P. Phosphorylation of the catalytic subunit of Na+,K(+)-ATPase inhibits the activity of the enzyme. Proceedings of the National Academy of Sciences. 1991;88(24):11359–11362. 10.1073/pnas.88.24.11359 PMC53134 1662394

[29] Rouch AJ, Kudo LH.Role of PGE (2) in alpha (2)-induced inhibition of AVP- and cAMP-stimulated H(2)O, Na(+), and urea transport in rat IMCD. Am J Physiol Renal Physiol. 2000; 279(2):F294–301. doi: 10.1152/ajprenal.2000.279.2.F294 10919849 10.1152/ajprenal.2000.279.2.F294

[30] Feleder C. Preoptic 1- and 2-noradrenergic agonists induce, respectively, PGE2-independent and PGE2-dependent hyperthermic responses in guinea pigs. AJP: Regulatory, Integrative and Comparative Physiology. 2004;286(6):R1156–R1166. 10.1152/ajpregu.00486.2003 14962823

[31] Averbeck B, Reeh P, Michaelis M. Modulation of CGRP and PGE2 release from isolated rat skin by α-adrenoceptors and α-opioid-receptors. Neuroreport. 2001;12(10):2097–2100. 11447314 10.1097/00001756-200107200-00011

[32] Skayian Y, Kreydiyyeh S. Tumor necrosis factor alpha alters Na+–K+ ATPase activity in rat cardiac myocytes: Involvement of NF-κB, AP-1 and PGE2. Life Sciences. 2006;80(2):173–180. doi: 10.1016/j.lfs.2006.08.037 17028035 10.1016/j.lfs.2006.08.037

[33] Kreydiyyeh SI, Riman S, Serhan M, Kassardjian A.TNF-alpha modulates hepatic Na+-K+ ATPase activity via PGE2 and EP2 receptors.Prostaglandins Other Lipid Mediat. 2007; 83(4):295–303 doi: 10.1016/j.prostaglandins.2007.02.003 17499749 10.1016/j.prostaglandins.2007.02.003

[34] Seven I., Türközkan N., & Çimen B. The effects of nitric oxide synthesis on the Na+,K+-ATPase activity in guinea pig kidney exposed to lipopolysaccharides. Molecular and Cellular Biochemistry. 2005; 271(1–2):107–112. 15881661 10.1007/s11010-005-5616-1

[35] Çimen B, Türközkan N, Seven I, Ünlü A, Karasu Ç. Impaired Na+, K+-ATPase activity as a mechanism of reactive nitrogen species-induced cytotoxicity in guinea pig liver exposed to lipopolysaccharides. Molecular and cellular biochemistry. 2004; 259: 53–57. 15124907 10.1023/b:mcbi.0000021344.64317.a2

[36] Irtegun S, Wood R, Ormsby A, Mulhern T, Hatters D. Tyrosine 416 Is Phosphorylated in the Closed, Repressed Conformation of c-Src. PLoS ONE. 2013;8(7):e71035 doi: 10.1371/journal.pone.0071035 23923048 10.1371/journal.pone.0071035 PMC3724807

[37] Bjorge J, Jakymiw A, Fujita D. Selected glimpses into the activation and function of Src kinase. Oncogene. 2000;19(49):5620–5635. 11114743 10.1038/sj.onc.1203923

[38] Chen YH, Pouysségur J, Courtneidge SA, Van Obberghen-Schilling E. Activation of Src family kinase activity by the G protein-coupled thrombin receptor in growth-responsive fibroblasts. J Biol Chem. 1994; 269(44):27372–7. 7525555

[39] Abrahamsen H, Vang T, Taskén K. Protein kinase A intersects SRC signaling in membrane microdomains. J Biol Chem. 2003; 278(19):17170–7 doi: 10.1074/jbc.M211426200 12606547 10.1074/jbc.M211426200

[40] Thobe BM, Frink M, Choudhry MA, Schwacha MG, Bland KI, Chaudry IH. Src family kinases regulate p38 MAPK-mediated IL-6 production in Kupffer cells following hypoxia. Am J Physiol Cell Physiol. 2006; 291(3):C476–82. doi: 10.1152/ajpcell.00076.2006 16571868 10.1152/ajpcell.00076.2006

[41] Pechkovsky D, Scaffidi A, Hackett T, Ballard J, Shaheen F, Thompson P et al Transforming Growth Factor β1 Induces αvβ3 Integrin Expression in Human Lung Fibroblasts via a β3 Integrin-, c-Src-, and p38 MAPK-dependent Pathway. Journal of Biological Chemistry. 2008; 283(19):12898–12908. doi: 10.1074/jbc.M708226200 18353785 10.1074/jbc.M708226200

[42] Frey M, Golovin A, Polk D. Epidermal Growth Factor-stimulated Intestinal Epithelial Cell Migration Requires Src Family Kinase-dependent p38 MAPK Signaling. Journal of Biological Chemistry. 2004;279(43):44513–44521. doi: 10.1074/jbc.M406253200 15316018 10.1074/jbc.M406253200

[43] Singh R, Dhawan P, Golden C, Kapoor G, Mehta K. One-way Cross-talk between p38MAPKand p42/44MAPK. Journal of Biological Chemistry. 1999;274(28):19593–19600. 10391894 10.1074/jbc.274.28.19593

[44] Liu Q. Protein phosphatase 2A-mediated cross-talk between p38 MAPK and ERK in apoptosis of cardiac myocytes. AJP: Heart and Circulatory Physiology. 2004; 286(6):H2204–H2212. 14962831 10.1152/ajpheart.01050.2003

[45] Chae H, Chae S, Reed J, Kim H. Salicylate Regulates COX-2 Expression Through ERK and Subsequent NF-κB Activation in Osteoblasts. Immunopharmacology and Immunotoxicology. 2004;26(1):75–91. 15106733 10.1081/iph-120029946

[46] Shih C, Bien M, Chiang L, Su C, Lin C, Chen B. Thrombin induces cyclooxygenase-2 expression via the ERK and NF-κB pathways in human lung fibroblasts. European Journal of Pharmacology. 2009; 618(1–3):70–75. doi: 10.1016/j.ejphar.2009.07.007 19616539 10.1016/j.ejphar.2009.07.007

[47] Xia Q, Hu Q, Wang H, Yang H, Gao F, Ren H et al Induction of COX-2-PGE2 synthesis by activation of the MAPK/ERK pathway contributes to neuronal death triggered by TDP-43-depleted microglia. Cell Death and Disease. 2015;6(3):e1702. 25811799 10.1038/cddis.2015.69 PMC4385945

[48] Jin XH, Wang ZQ, Siragy HM, Guerrant RL, Carey RM. Regulation of jejunal sodium and water absorption by angiotensin subtype receptors. Am J Physiol. 1998; 275(2 Pt 2):R515–23. 9688688 10.1152/ajpregu.1998.275.2.R515

[49] Kreydiyyeh S, Al-Sadi R. The signal transduction pathway that mediates the effect of interleukin-1 beta on the Na + -K + -ATPase in LLC-PK 1 cells. Pflugers Archiv European Journal of Physiology. 2004; 448(2):231–238. doi: 10.1007/s00424-004-1242-0 14985981 10.1007/s00424-004-1242-0

[50] Oliveira MS, Furian AF, Rambo LM, Ribeiro LR, Royes LF, Ferreira J, Calixto JB, Otalora LF, Garrido-Sanabria ER, Mello CF. Prostaglandin E2 modulates Na+,K+-ATPase activity in rat hippocampus: implications for neurological diseases. J Neurochem. 2009; 109(2):416–26. doi: 10.1111/j.1471-4159.2009.05961.x 19200345 10.1111/j.1471-4159.2009.05961.x

